# Microbiome profiling reveals gut bacterial species associated with rapid lung function decline in people with HIV

**DOI:** 10.3389/fimmu.2025.1555441

**Published:** 2025-06-10

**Authors:** Xiangning Bai, Sajan C. Raju, Andreas Dehlbæk Knudsen, Rebekka Faber Thudium, Nicoline Stender Arentoft, Marco Gelpi, Safura-Luise Heidari, Ken M. Kunisaki, Karsten Kristiansen, Johannes Roksund Hov, Susanne Dam Nielsen, Marius Trøseid

**Affiliations:** ^1^ Department of Microbiology, Division of Laboratory Medicine, Oslo University Hospital, Oslo, Norway; ^2^ Center for Infectious Medicine, Department of Medicine Huddinge, Karolinska Institutet, Stockholm, Sweden; ^3^ Research Institute of Internal Medicine, Oslo University Hospital, Oslo, Norway; ^4^ Institute of Clinical Medicine, University of Oslo, Oslo, Norway; ^5^ Department of Infectious Diseases, Rigshospitalet, University of Copenhagen, Copenhagen, Denmark; ^6^ Department of Infectious Diseases, Hvidovre Hospital, University of Copenhagen, Hvidovre, Denmark; ^7^ Section of Pulmonary, Allergy, Critical Care and Sleep Medicine, Minneapolis Veterans Affairs Health Care System, Minneapolis, Minnesota, MN, United States; ^8^ Division of Pulmonary, Allergy, Critical Care and Sleep Medicine, University of Minnesota, Minneapolis, Minnesota, MN, United States; ^9^ BGI-Shenzhen, Shenzhen, China; ^10^ Laboratory of Integrative Biomedicine, Department of Biology, University of Copenhagen, Copenhagen, Denmark; ^11^ Norwegian PSC Research Center, Department of Transplantation Medicine, Oslo University Hospital, Oslo, Norway; ^12^ Department of Clinical Medicine, Faculty of Health and Medical Sciences, University of Copenhagen, Copenhagen, Denmark; ^13^ Department of Surgical Gastroenterology, Rigshospitalet, Copenhagen University Hospital, Copenhagen, Denmark; ^14^ Section of Clinical Immunology and Infectious Diseases, Department of Rheumatology, Dermatology and Infectious Diseases, Oslo University Hospital, Oslo, Norway

**Keywords:** HIV, gut microbiome, pulmonary comorbidity, lung function decline, airflow limitation, spirometry

## Abstract

**Background:**

People with HIV (PWH) have an increased risk of pulmonary comorbidities compared to people without HIV. The gut microbiome regulates host immunity and is altered in PWH. This study aims to determine potential associations between gut microbiome, lung function decline, and airflow limitation in PWH.

**Methods:**

PWH from the Copenhagen Comorbidity in HIV Infection (COCOMO) Study with available lung function testing and microbiome data were included (n=385). The gut microbiome was characterized using shotgun metagenomic sequencing. Associations between gut microbiome, rapid lung function decline, and airflow limitation were analysed in multivariable logistic regressions adjusted for traditional and HIV-associated risk factors for lung disease.

**Results:**

Several bacterial species were significantly enriched in PWH with rapid lung function decline, including opportunistic pathogenic bacterial species *Bacteroides coprophilus*, *Klebsiella michiganensis*, and *Clostridium perfringens*. A gut microbial dysbiosis index based on compositional changes was associated with rapid lung function decline (adjusted odds ratio (aOR) 1.18, 95% confidence interval (CI) [1.11-1.27], p<0.001), and airflow limitation (aOR 1.16, 95% CI [1.04-1.29], p=0.007) in adjusted multivariable logistic regression analyses.

**Conclusion:**

Associations between the gut dysbiosis index and rapid lung function decline and airflow limitation suggest a potential role of certain gut bacterial species in the pathogenesis of pulmonary comorbidities in PWH.

## 1 Introduction

PWH, even well-treated, have a higher risk of chronic pulmonary comorbidities compared to the background population, in particular chronic obstructive pulmonary disease (COPD) (1, 2). In fact, chronic lung disease was the most prevalent comorbidity in a recent study investigating trends in non-AIDS related comorbidities over a 10-year period in hospitalized PWH in New York (3), and respiratory mortality is one of the few causes of death in PWH that has not declined over the last 20 years (4).

Importantly, a Dutch study showed a decline in forced expiratory volume in one second (FEV_1_) and forced vital capacity (FVC) in PWH compared with controls (5). Likewise, a recent work from the COCOMO study showed that even well-treated PWH with no history of smoking had faster lung function decline than population-based controls (2). Yet, the underlying mechanisms involved in the pathogenesis of pulmonary comorbidities and faster lung function decline in PWH are poorly understood. Risk factors may include smoking, which is more prevalent in PWH than in the background population, older age, history of pneumonia, oxidative stress, immune cell activation, and chronic airway inflammation (6). Indeed, increased eosinophilic airway inflammation, as measured by higher exhaled nitric oxide level, was observed in PWH in the COCOMO study (7). Furthermore, a recent study showed that elevated interleukin (IL)-1β and IL-10 were independently associated with faster lung function decline in PWH, suggesting that dysregulated systemic inflammation may play a role in the pathogenesis of pulmonary comorbidities (8).

The human gut microbiome has a fundamental impact on immune system and inflammation, contributing to disease or exacerbating pre-existing conditions (9). Recent studies, including our own (10, 11), have shown that the gut microbiome and its metabolic by-products differ in PWH versus controls, even after adjustment for known confounders such as age and sexual orientation, and that these alterations correlate with previous immunodeficiency and present chronic inflammation (10). Changes in the gut microbiome may contribute to pulmonary diseases through effects on systemic inflammation, microbial translocation, or microbial metabolites via a potential gut-lung axis (12, 13). Dysbiosis in the respiratory microbiome has in previous studies been linked to increased risks of adverse pulmonary outcomes in PWH (14, 15). However, little is known about the association between gut microbiome, pulmonary disease, and lung function decline in PWH, as well as the potential of gut-lung microbiome connections in the HIV-associated pulmonary disease.

The primary aim of this study was to determine the potential associations between the gut microbiome including microbial species and their pathways, and lung function decline in PWH. The secondary aim was to determine the associations between the gut microbiome and airflow limitation as defined by Global Initiative for Chronic Obstructive Lung Disease (GOLD) (16).

## 2 Materials and methods

### 2.1 Study design and participants

The Copenhagen Comorbidity in HIV Infection (COCOMO) study is an ongoing longitudinal cohort study designed to determine the burden of, and risk factors for, non-AIDS comorbidities in PWH in Copenhagen, Denmark (17). Spirometry was performed at baseline from March 2015 until November 2016, and at subsequent follow-up visits between April 2017 and April 2019 with a median follow-up time of 2.3 years, as described previously (2). Eligibility criteria for this study were participants in COCOMO aged ≥25 years with two valid spirometry tests separated by at least 2 years of follow-up. Only PWH who had received antiretroviral therapy (ART) for a minimum of 6 months at baseline and had available fecal samples were included in the present study. Participants who provided fecal samples were older than participants who did not provide fecal samples (53 *vs* 49 years old, p<0.001), but they did not differ on any of the other parameters when adjusted for age (all p>0.05).

### 2.2 Lung function testing

Lung function was assessed by spirometry using an ultrasonic spirometer (EasyOne, ndd Medical, Zürich, Switzerland). Prebronchodilator forced expiratory volume in 1 second (FEV_1_) and forced vital capacity (FVC) were measured at baseline and at follow-up in accordance with the European Respiratory Society/American Thoracic Society recommendations (18). Predicted values for FEV_1_ and FVC, along with z-scores and the lower limit of normal for the FEV_1_/FVC ratio, were determined using the multi-ethnic reference equations from the Global Lung Function Initiative (19). Lung function decline was measured as changes in FEV_1_ milliliter (mL) per year (8). In relation to each test, a quality grading from A to F was automatically generated by the EasyOne spirometer, spirometry data with grade A-C were defined as high quality and were used for sensitivity analyses.

### 2.3 Outcomes definition

The primary outcome was rapid lung function decline defined as FEV_1_ decline >40 mL/year as previously described (2). The secondary outcome was airflow limitation defined as FEV_1_/FVC <0.70 and FEV_1_ <80% of the predicted value corresponding to COPD of moderate or worse severity (≥2 COPD grade) according to the GOLD (16).

### 2.4 Inflammatory markers measurements

Plasma concentrations of pro- and anti-inflammatory cytokines were measured using Luminex immunoassays (R&D systems, Minneapolis, Minnesota), as previously described in detail (8). In brief, the multiplex assay kits included the proinflammatory markers interleukin 1-beta (IL-1β), interleukin 2 (IL-2), IL-6, interferon gamma (IFN-γ), and tumor necrosis factor alpha (TNF-α) and the anti-inflammatory marker interleukin 10 (IL-10). The concentrations of microbial translocation marker soluble CD14 (sCD14) and monocyte activation marker soluble CD163 (sCD163), were measured in plasma by high sensitivity enzyme-linked immunosorbent assay kits (R&D systems, Minneapolis, Minnesota) at the Research Institute of Internal Medicine, Division of Surgery, Inflammatory Diseases and Transplantation, Oslo University Hospital, Rikshospitalet, Oslo, Norway. High-sensitivity CRP (hs-CRP) was measured using a turbidimetric assay at the Department of Clinical Biochemistry, Herlev Hospital, Copenhagen, Denmark.

### 2.5 Fecal sample collection and processing

At study inclusion, participants were instructed to collect fecal samples using a standardized sampling device and collection tubes with DNA Stabilizer (Stratec Molecular). Samples were frozen at −80°C on arrival and eventually shipped on dry ice to Oslo for microbiome analyses. DNA was extracted using the PSPSpin Stool DNA-Plus Kit (Stratec Molecular), following the manufacturer’s protocol, slightly modified by adding a bead-beating step, as described elsewhere (10).

### 2.6 Shotgun metagenome sequencing and microbiome profiling

In the COCOMO study, which included 1099 participants, 385 individuals had both lung function measurements and fecal samples collected at baseline. Shotgun metagenome sequencing was performed on these samples using 150 bp paired-end sequencing on the MGISEQ-T7 platform at MGI Tech, Mārupe, Latvia. The pre-processing of the raw reads was conducted using the *KneadData* pipeline (v0.10.0). Initially, raw reads were trimmed to a *Phred* score of 20 and reads below the specified minimum length were removed using *Trimmomatic* (version 0.39), which was integrated within the *KneadData* pipeline (20). Subsequently, trimmed reads were aligned against the human reference genome using bowtie2 (21), reads aligning to the human genome were eliminated from further analysis. Taxonomic profiling was performed using MetaPhlAn (v3.0.0.0) by default including reference genomes of bacteria, archaea, and eukaryotes (22). HUMAnN3 (v3.0.0) (22) was employed for functional profiling (genes and pathways), incorporating MetaPhlAn, DIAMOND 0.9.36 (23), and the databases uniref90 (v201901) (24) and mpa_v30_CHOCOPhlAn_201901. Additionally, metabolic pathways were identified from the MetaCyc database (metacyc.org).

Taxonomic profiles obtained from MetaPhlAn were imported and analyzed using the phyloseq v1.40.0 (25) package in R. To evaluate taxonomic and functional richness as well as diversity, several alpha diversity indices including the Shannon index and inverse Simpson index were employed. Beta-diversity was assessed by calculating the Bray–Curtis dissimilarity index. Ordinations were constructed using Principal Coordinate Analysis (PCoA). PERMANOVA was performed with adjustment for the significantly different covariates between groups (**Table 1**), using the vegan::adonis2 function between the groups with 999 permutations. Benjamini-Hochberg (BH) method was used to control the false discovery rate for multiple comparisons.

**Table 1:** Demographic characteristics of PWH in present sub-study of COCOMO.

Characteristic	Rapid lung function decline (FEV_1_ > 40 mL/year)		Airflow limitation	
	No, n = 192*^1^*	Yes, n= 155*^1^*	No, n = 348*^1^*	Yes, n = 37*^1^*
**Age**, years	53.3 (10.9)	53.3 (10.7)	52.5 (10.5)	61.8 (10.5)*
**Sex,** male	162 (84%)	135 (87%)	275 (87%)	22 (73%)
**BMI**, kg/m^2^	24.6 (3.6)	24.9 (3.9)	24.8 (3.7)	23.6 (4.8)*
Smoking status				
Current smoker	40 (21%)	42 (28%)*	69 (22%)	13 (45%)*
Former smoker	79 (42%)	59 (40%)*	124 (40%)	14 (48%)*
Never smoker	71 (37%)	48 (32%)*	117 (38%)	2 (6.9%)*
**HIV duration**, years	16.0 (9.1)	15.5 (9.3)	15.2 (9.1)	21.8 (8.5)**
Ethnicity				
Caucasian	171 (89%)	136 (88%)	279 (88%)	28 (93%)
Other	21 (11%)	19 (12%)	38 (12%)	2 (6.7%)
**IL-1β** ^§^, pg/mL	0.2 (0.2)	0.2 (0.2)	0.2 (0.2)	0.2 (0.2)
**IL-10** ^§^, pg/mL	0.9 (1.5)	0.7 (0.6)	0.8 (1.1)	1.2 (1.7)
**CD4 count,** cells/mm^3^	706.1 (278.1)	719.1 (262.3)	708.7 (273.1)	744.4 (261.9)
**Nadir CD4** count <200 cells/µL	72 (21.6%)	61 (17.6%)*	125 (32.5%)	11 (2.9%)
**cART time**, years	12.0 (6.5)	11.7 (6.8)	11.4 (6.6)	15.6 (6.2)**
**HIV RNA at baseline <**50 copies/mL	186 (97%)	148 (95%)	304 (96%)	30 (100%)

Continuous data as mean (standard deviation), *p<0.05, **p<0.001

*^1^* n (%)

^§^None of the inflammatory markers (described in Methods) were statistically different between groups including IL-1β and IL-10, which were however presented and included in multivariable logistic regression model as they were associated with faster lung function decline in the total cohort (8).

Differential abundance (DA) analyses were conducted to test the differences in the relative abundance in microbial taxa, genes, and metabolic pathways between groups, using the DESeq2 package with the Wald test and parametric fitting (26), and further adjusted for smoking status, which was associated with rapid lung function decline as well as airflow limitation (**Table 1**). Log2 (fold-change) and BH-adjusted p values (< 0.05) were extracted for each comparison from the model. Analyses were carried out at different taxonomic (i.e., species, genus, and family), and functional (i.e., genes and pathways) levels. Based on differentially abundant microbial species from the DESeq2 analysis, a microbial dysbiosis index (quantitative measure used to assess the balance or imbalance of microbial communities), was calculated as log10 (sum of the abundances of the species increased in rapid decliners/sum of the abundances of species decreased in rapid decliners), as first reported by Gevers et al. (27), and in accordance with other microbiome studies from the COCOMO cohort (10, 28). In addition, Mann–Whitney U test was performed further as a sensitivity analysis of differentially abundant species identified by DESeq2, and taxa with a p-value >0.10 were removed from the dysbiosis index.

### 2.7 Statistics

Descriptive statistics were presented by expressing continuous variables as mean ± standard deviation and categorical variables as numbers and percentages. Differences in distributions of continuous variables were tested using Mann–Whitney U tests. DA analyses in DESeq2 were adjusted for significant covariate smoking status at follow-up (current, former, never) for both the primary outcome (rapid lung function decline), and the secondary outcome (airflow limitation).

Associations between the microbial dysbiosis index and primary outcome was further adjusted for other traditional (age, sex, BMI, ethnicity) and HIV-related (transmission mode, nadir CD4 count, HIV duration) risk factors for lung disease in model 1, using multivariable logistic regressions, and further adjusted for soluble levels of IL-1β and IL-10 (previously associated with rapid lung function decline in PWH (8)) in model 2, to verify if the microbial dysbiosis index associated with rapid lung function decline was robust and independent of these potential confounders. Moreover, a sensitivity analysis was performed by excluding those with self-reported history of pneumonia in the decade prior to fecal sampling to rule out its potential confounding effect on rapid lung function decline.

We further tested if the microbial dysbiosis index was also associated with the secondary outcome airflow limitation. Due to the low sample size of airflow limitation, association between the dysbiosis index and airflow limitation was separately adjusted for age, BMI, and a third significant covariate (**Table 1**) at a time.

Spearman’s correlation analysis was performed between differentially abundant species from DESeq2 analysis and HIV-related clinical parameters, and visualized by the ggcorrplot (Ver 0.1.4.1) package in R.

The co-occurrence network plots were generated for rapid decliners and normal decliners using the iGraph R package (V 2.1.1) using the subset of top abundant taxonomy profiles, including the differentially abundant taxa from DESEq2 analysis, from MetaPhlAn3 to assess the correlations among microbial taxa based on their Spearman’s correlations.

## 3 Results

### 3.1 Baseline characteristics of PWH with lung function abnormalities

Among 385 participants who had lung function measurement and metagenome data at baseline, 347 had valid lung function data also at follow-up, thus had measured FEV_1_ decline, and 155 (44.7%) presented with rapid lung function decline (**Table 1**). PWH with rapid lung function decline were overrepresented by current smokers, whereas airflow limitation was associated with current smoking, older age, lower BMI, longer HIV duration and cART time.

### 3.2 Association between gut microbiome and rapid lung function decline in PWH

**Figure 1:**
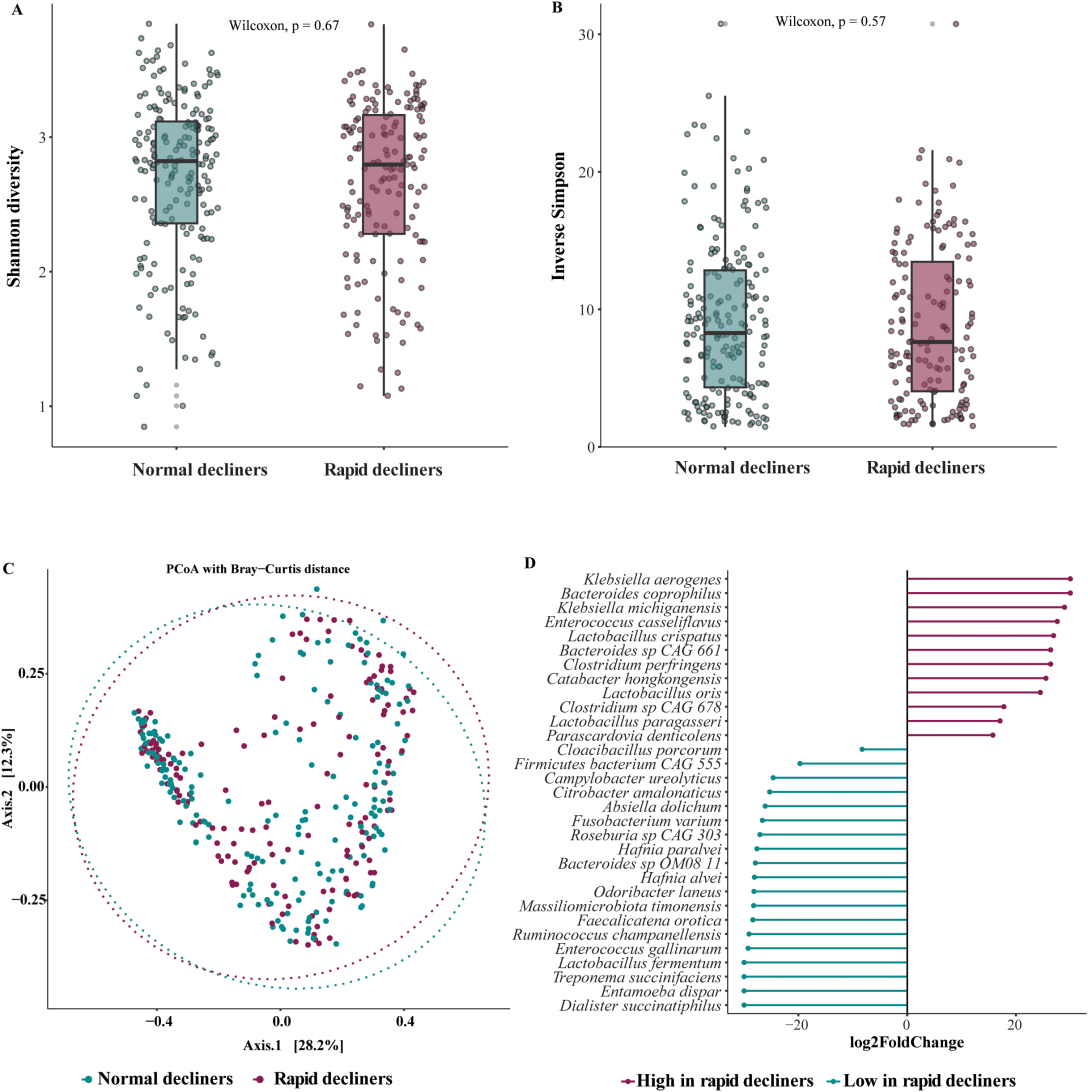
Gut microbiome profiles at species level in PWH with and without rapid lung function decline. **(A)**, Shannon diversity index. **(B)**, inverse Simpson diversity index. **(C)**, Beta-diversity assessed by the Bray–Curtis dissimilarity index, visualized using Principal Coordinate Analysis (PCoA). **(D)**, Bacterial species differentially abundant between PWH with and without rapid lung function decline, analyzed using the DESeq2 package, adjusted for smoking.

No statistically significant difference in microbial alpha diversity at species level measured by Shannon and inverse Simpson index was observed between PWH with and without rapid lung function decline (**Figures 1A, B**). No difference in microbiome composition at species level (beta diversity) was observed between the two groups (**Figure 1C**). Similarly, no difference in microbial alpha or beta diversity was observed at genus or family level (**Supplementary Figures S1A–E, G**).

On the compositional level, after adjusting for smoking status (current, former, never) in DESeq2, several bacterial species were significantly enriched in PWH with rapid lung function decline compared to those with normal lung function decline (**Figure 1D**). After removing non-significant taxa from Mann Whitney U test, species enriched in PWH with rapid lung function decline included *Bacteroides coprophilus*, *Klebsiella michiganensis*, *Catabacter hongkongensis*, *Clostridium perfringens*, *Lactobacillus oris*, *Lactobacillus paragasseri*, while the relative abundance of two species, *Cloacibacillus porcorum* and *Absiella dolichum* were decreased in rapid decliners. These differentially abundant species between rapid and normal lung function decliners were used to define a microbial dysbiosis index as previously described (27), and explained in the Methods section. At the genus level, the relative abundances of *Helicobacter*, *Catabacter*, and *Parascardovia* were significantly enriched, while the relative abundances of 6 genera were decreased in rapid decliners compared to normal decliners (**Supplementary Figure S1F**). At the family level, the relative abundance of *Helicobacteraceae* was significantly increased, while the relative abundance of *Spirochaetaceae*, *Entamoebidae*, *Hafniaceae* were decreased in rapid decliners (**Supplementary Figure S1H**).

At the functional level, no significant difference in gene richness or diversity was found between rapid and normal decliners (**Supplementary Figures S2A–C**). No difference in alpha or beta diversity in pathways was found between the two groups (**Supplementary Figures S3A–C**). No differentially abundant genes or pathways were found between rapid and normal decliners after adjusting for smoking status (current, former, never) at follow-up.

### 3.3 Gut microbiome profiles in PWH with and without airflow limitation

No statistically significant difference was observed in microbial alpha or beta diversity between PWH with and without airflow limitation (**Supplementary Figures S4A–C**). After adjusting for smoking status, the relative abundances of five species, including *Bacteroides* sp *CAG 661* which was also enriched in rapid decliners, were significantly enriched in PWH with airflow limitation, while the relative abundances of several species were decreased in this group (**Supplementary Figure S4D**).

### 3.4 Prediction of rapid lung function decline by gut microbial dysbiosis

Finally, we assessed whether gut microbial dysbiosis could predict rapid lung function decline independently of relevant confounders in addition to smoking status. In a multivariable logistic regression adjusted for other traditional (age, sex, BMI, ethnicity) and HIV-related (transmission mode, nadir CD4 count, HIV duration) risk factors for lung disease, the dysbiosis index was independently associated with rapid lung function decline (aOR 1.18, 95% CI [1.11-1.27], p<0.001). Restricting analyses to PWH with no self-reported history of pneumonia (62%) did not alter the association between microbial dysbiosis index and rapid lung function decline (aOR 1.25, 95% CI [1.14-1.38], p<0.001). Furthermore, restricting analyses to PWH with predefined high-quality spirometry data (86.2%) did not alter the association between microbial dysbiosis index and rapid lung function decline either (aOR 1.16, 95% CI [1.09-1.25], p<0.001).

The microbial dysbiosis index was not associated with levels of inflammatory markers (described in Methods), and adjusting further for IL-1β and IL-10 which were associated with faster lung function decline in the total cohort (8) did not alter the association between microbial dysbiosis index and rapid lung function decline.

Importantly, the microbial dysbiosis index based on rapid decliners was associated with airflow limitation in multivariable analyses adjusted for significant confounders for airflow limitation (age, BMI, HIV duration, and cART time) (aOR 1.16, 95% CI [1.04-1.29], p=0.007). No significant correlations between microbial taxa within the dysbiosis index (i.e., differentially abundant taxa between rapid and normal decliners) and clinical parameters were found, with a few exceptions showing weak correlations (Spearman’s correlation coeffecient < ±0.2), though correlations were observed between some clinical parameters as anticipated (**Supplementary Figure S5**).

A number of microbial taxa were closely correlated in co-occurrence plots (Spearman’s correlation coeffecient>0.5 and p<0.05) both in rapid and normal decliners (**Supplementary Figure S6**), while no significant correlations were observed among most taxa within the microbial dysbiosis index (**Supplementary Figure S6**, **Supplementary Figure S5**).

## 4 Discussion

In this study using a large cohort of well-treated PWH, we found no significant difference in overall gut microbial diversity between PWH with and without rapid lung function decline. However, several microbial species were differentially abundant between PWH with and without rapid lung function decline after adjustment for smoking status. After further adjustment for traditional and HIV-related risk factors for lung disease, as well as cytokines (IL-1β, IL-10), the gut microbial dysbiosis index was independently associated with rapid lung function decline. Importantly, we also found an independent association of the gut microbial dysbiosis index with the airflow limitation in PWH, suggesting that the gut dysbiosis based on the lung function decline may be predictive for airflow limitation. Moreover, we found no overlap at bacterial genus or family levels between microbial dysbiosis associated with rapid lung function decline among PWH in this study and our previously defined HIV-related microbiome dysbiosis using 16S rRNA gene sequencing (10), suggesting that microbiome dysbiosis-associated rapid lung function decline is not related to HIV status per se.

We observed that some opportunistic pathogenic bacterial species were significantly enriched in PWH with rapid lung function decline compared to those without, e.g., *Bacteroides coprophilus*, *Klebsiella michiganensis, Clostridium perfringens. Bacteroides coprophilus* has been associated with COVID-19 and multisystem inflammatory syndrome (29). *Klebsiella michiganensis*, among other *Klebsiella* species, has been reported to be dominant in gut microbiota among infants with lower respiratory infection (30). *Clostridium perfringens* produces severe and rapidly fatal enterotoxemia affecting several organs including lungs, and a previous study showed that lung endothelial cells are sensitive to epsilon toxin from *C. perfringens* (31). The enrichment of these pathogenic bacterial species may play a role in the pulmonary pathology in PWH and contribute to rapid lung function decline, either due to the gut microbiome functioning as a reservoir for the lung microbiome, or through an indirect effect mediated by e.g., systemic inflammation caused by gut microbiota dysbiosis, yet the underlying mechanisms and causal link are to be elucidated.

We investigated further if the microbial dysbiosis index was correlated with soluble levels of IL-1β and IL-10, which in our previous work were associated with rapid lung function decline in the total COCOMO cohort (8). However, the association between the microbial dysbiosis index and lung function decline was not altered when adjusting for these markers. This may indicate that pulmonary pathological effect may be more driven by other mechanisms linked with gut microbial dysbiosis than systemic inflammation. For instance, gut microbiome may serve as a reservoir for the airway microbiome, the dysbiosis in the gut could lead to airway microbiome dysbiosis, which directly contribute to the pulmonary pathology. Further studies on airway microbiome in correlation to pulmonary health are required.

We observed no significant difference in the gut microbiome diversity between PWH with and without airflow limitation. Nevertheless, increased relative abundance of several species were found in PWH with airflow limitation. Of a potential interest, *Bacteroides* sp *CAG 661* was also associated with rapid lung function decliner, suggesting its pathogenic potential to the lung. It should be acknowledged that our sample size regarding airflow limitation at baseline is a major limitation, the observed correlations are to be further validated in future studies.

Our findings are in line with two previous studies of cohorts including PWH and HIV-uninfected controls (32, 33). Both studies, using 16S rRNA gene sequencing, reported no associations between gut microbiota diversity and HIV status or lung function, while one study (32) showed that alterations in the oral microbiome were associated with impaired pulmonary function and systemic inflammation in PWH, but not among HIV-uninfected individuals. Of note, our study provides novel data on the potential role of several bacterial species in the rapid lung function among PWH, which has not been observed in the two earlier studies. This may be due to the variations in the study populations and different microbiome profiling methods. As it is known that 16S rRNA gene-based microbiome profiling detects only part of the microbiome community revealed by shotgun metagenome sequencing, and the less abundant taxa, particularly specific species detected only by shotgun sequencing can be biologically meaningful (34). In the general population, associations between gut microbiome and lung function decline have been reported, particularly in humans with COPD (35). For example, Bowerman et al. found that *Bifidobacteriaceae*, *Eubacteriaceae*, *Lactobacillaceae*, *Micrococcaceae*, *Streptococcaceae*, and *Veillonellaceae* were associated with COPD; while *Desulfovibrionaceae*, *Bacteroidaceae*, *Gastranaerophilaceae*, and *Selenomonadaceae* were negatively associated with COPD (36). We did not find overlap of bacteria associated with lung function decline in PWH identified in this study, and those commonly reported in the general population. Again, comparisons between different studies should be cautious and consider the variations in populations and microbiome approaches.

We acknowledge limitations and strengths of the study. First, we did not include HIV-negative controls due to lack of HIV-negative individuals in the COCOMO controls with matched microbiome samples and lung function data, further studies using shotgun metagenome sequencing and including both PWH and HIV-negative controls are thus warranted to validate our findings and to assess whether such findings are specific to PWH or not. Second, the sample size of PWH with airflow limitation at baseline was small; thus, the study was underpowered to draw robust conclusions regarding associations between gut microbiome and airflow limitation, larger validation cohorts are needed in future studies. Related to this limitation, our spirometry measures were pre-bronchodilator only, and therefore, we cannot determine how many of participants may have normalized lung function after bronchodilation (suggestive of asthma) versus had persistent airflow limitation (suggestive of COPD). We hence used the term ‘airflow limitation’ rather than COPD. Third, our results do not allow for determination of causal directionality. Furthermore, we acknowledge the lack of virome and resistome analysis from metagenome data, which is however not the focus of this study. The strengths of this study include the large sample size of PWH who had lung function measurements with longitudinal follow-up, and adjustments for a number of known confounders.

## 5 Conclusions

We report no significant changes in the overall gut microbiome composition and functional potential related to pulmonary comorbidity in PWH. However, changes in the relative abundances of several bacterial species were associated with rapid lung function decline, and the microbial dysbiosis index was independently associated with rapid lung function decline and airflow limitation. Future studies are warranted to validate our findings. In addition, there is a need of studies focusing on the airway microbiome, which may play a more important and more direct role in pulmonary comorbidities in PWH.

## Data Availability

The anonymised shotgun metagenome sequencing datasets presented in this study are deposited in the NCBI Sequence Read Archive (SRA) under the BioProject ID PRJNA1267518. Metadata are subject to restrictions due to approvals from the ethical committee and the Danish Data Protection Agency and cannot be shared publicly. Access to the metadata may be granted upon reasonable request to Dr. Susanne Dam Nielsen (Susanne.Dam.Poulsen@regionh.dk).
