# Supplementary material for: Microbiome profiling reveals gut bacterial species associated with rapid lung function decline in people with HIV
Source: Front Immunol. 2025 Jun 10;16:1555441. doi: 10.3389/fimmu.2025.1555441 (PMC12185991; doi:10.3389/fimmu.2025.1555441)
Supplement: Supplementary file 1 [file Table1.docx]

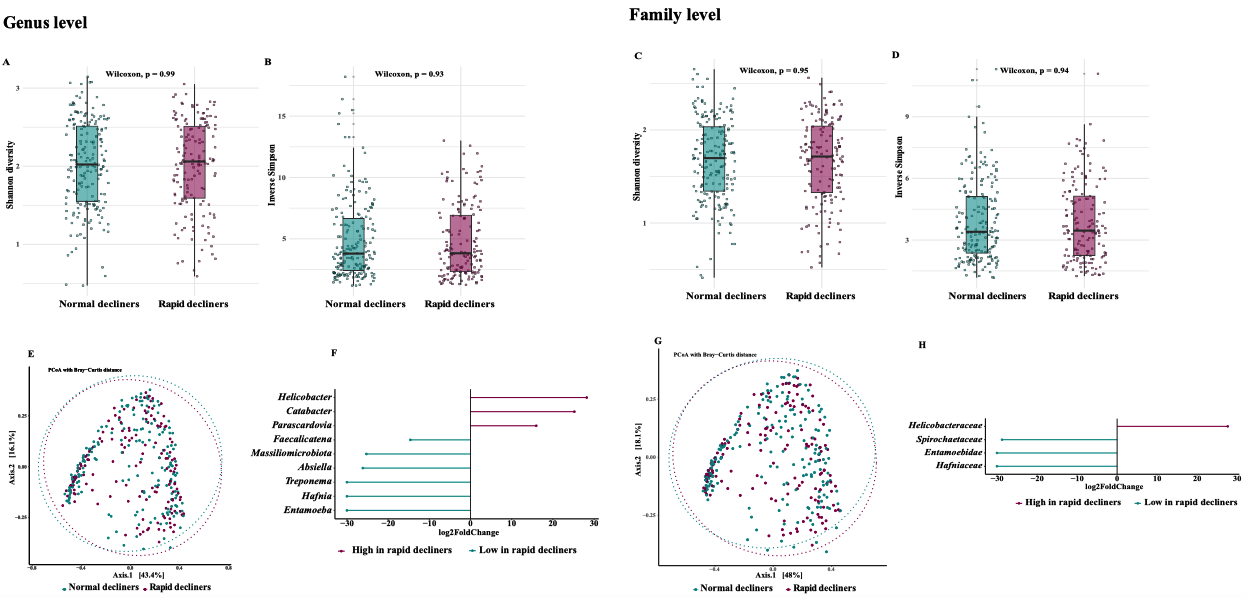


Supplementary **Figure S1**. Microbiome composition and diversity at genus and family level between PWH with and without rapid lung function decline. A-D, alpha diversity measured by Shannon diversity index and inverse Simpson diversity index, as indicated. E and G, Beta-diversity assessed by the Bray–Curtis dissimilarity index, visualized using Principal Coordinate Analysis (PCoA). Differentially abundant taxa at genus (F) and family (H) level between PWH with and without rapid lung function decline, analyzed using the DESeq2 package, adjusted for smoking status.


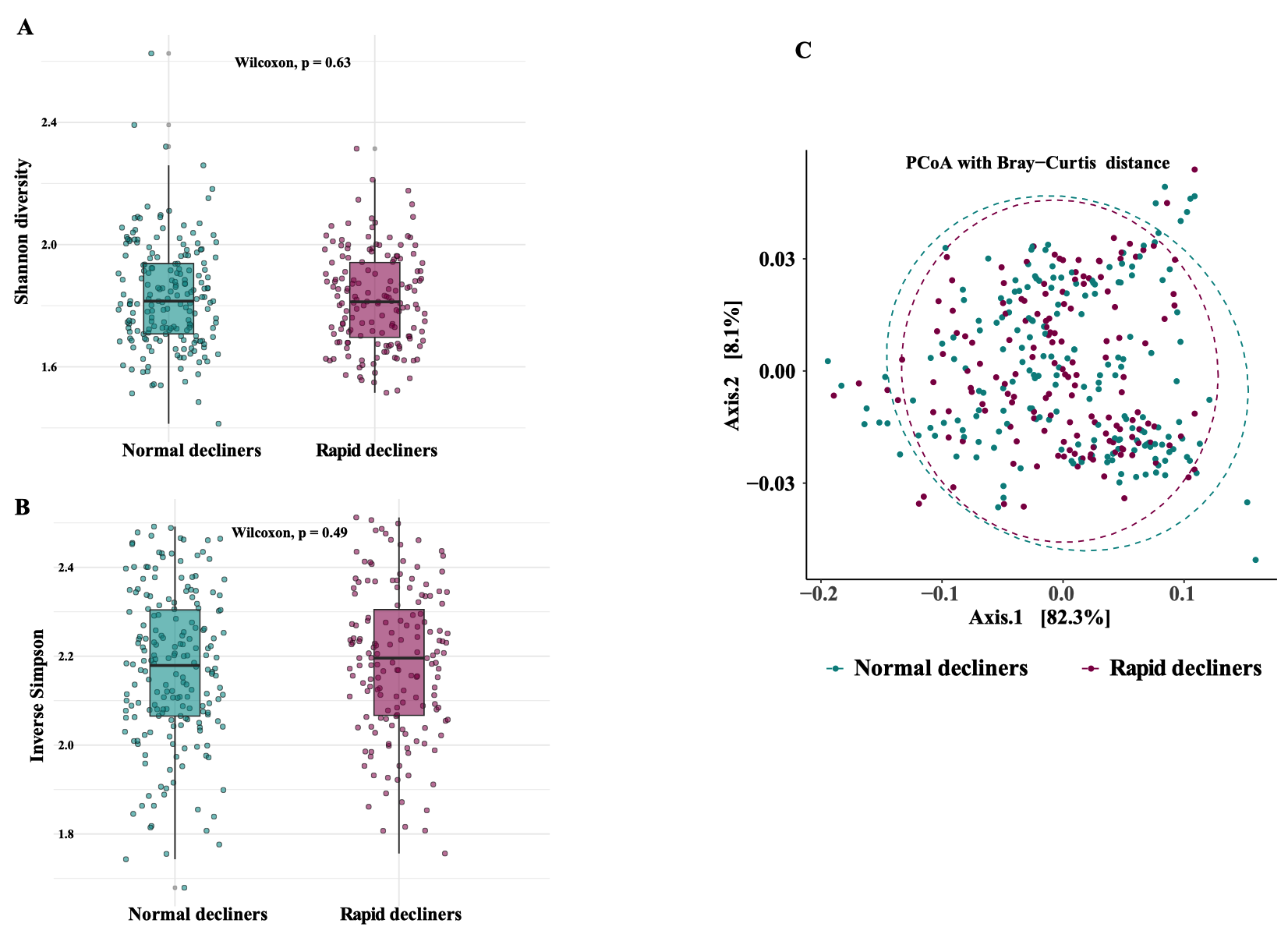


Supplementary **Figure S2.** Microbial functional gene richness and diversity between PWH with and without rapid lung function decline. A, Shannon diversity index. B, inverse Simpson diversity index. C, Beta-diversity assessed by the Bray–Curtis dissimilarity index, visualized using Principal Coordinate Analysis (PCoA).


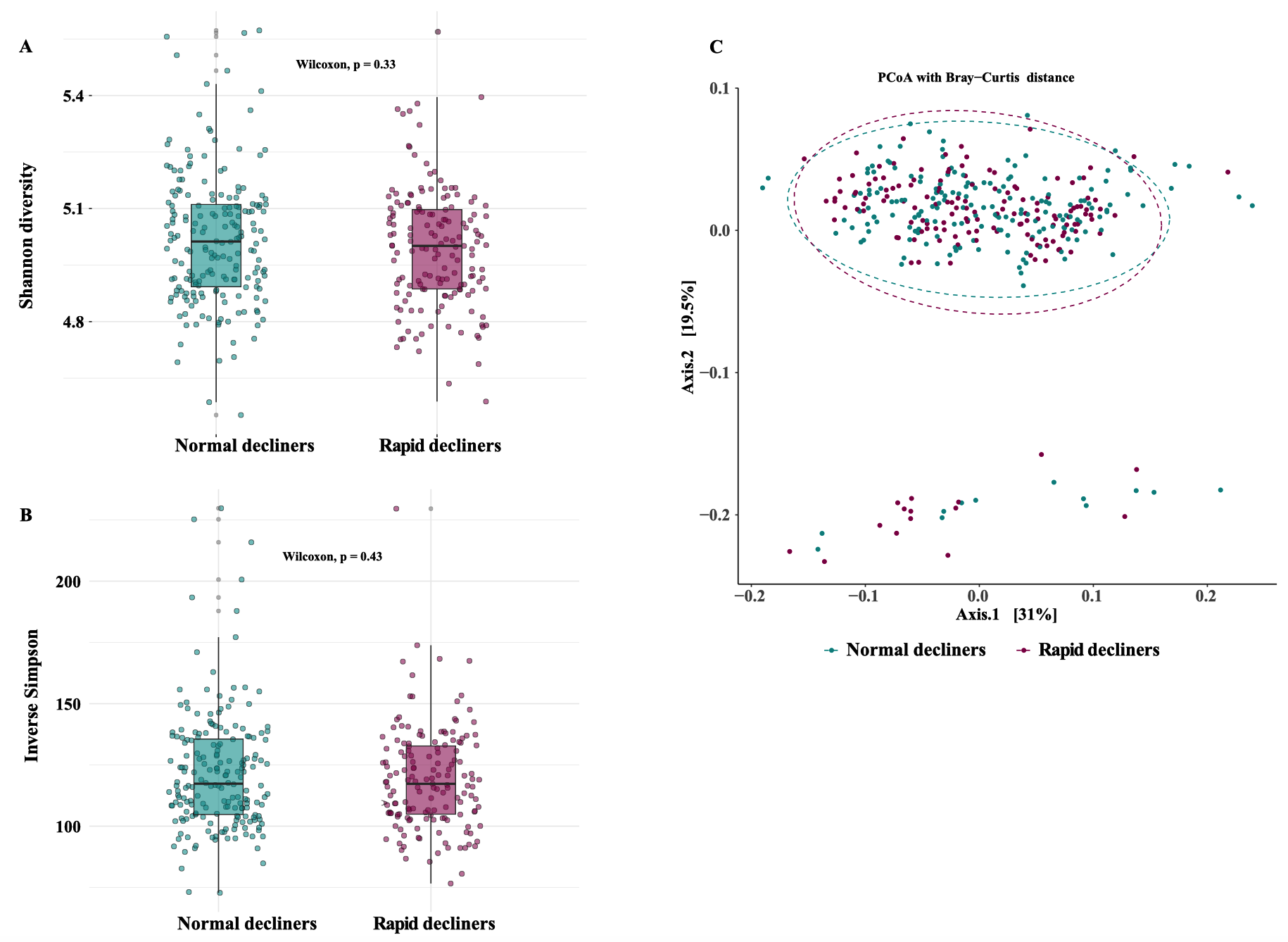


Supplementary **Figure S3.** Microbial functional pathways diversity between PWH with and without rapid lung function decline**.** A, Shannon diversity index. B, inverse Simpson diversity index. C, Beta-diversity assessed by the Bray–Curtis dissimilarity index, visualized using Principal Coordinate Analysis (PCoA).


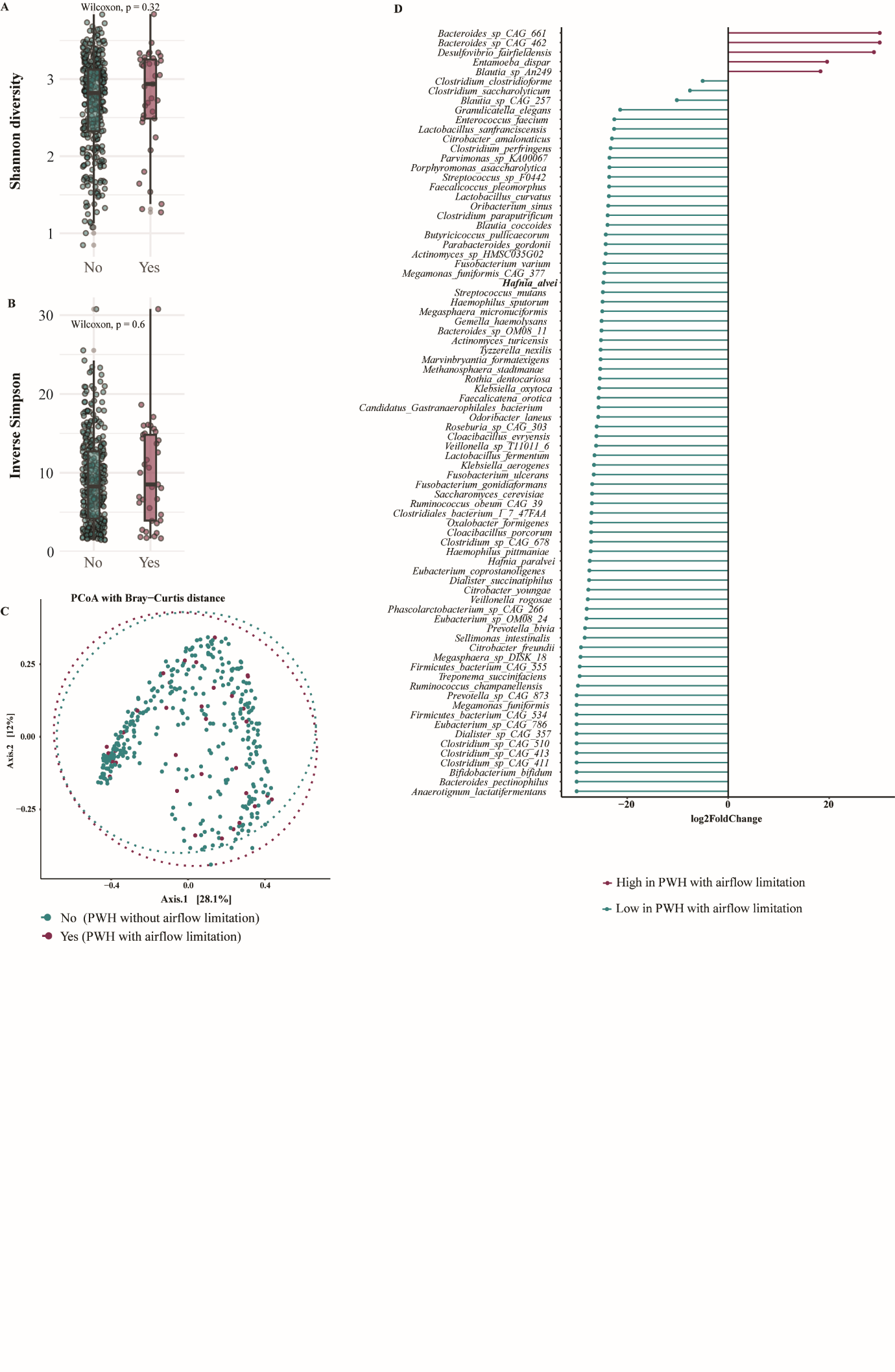


Supplementary **Figure S4**. Gut microbiome profiles in PWH with and without airflow limitation. A, Shannon diversity index. B, inverse Simpson diversity index. C, Beta-diversity assessed by the Bray–Curtis dissimilarity index, visualized using Principal Coordinate Analysis (PCoA). D, Microbial species differentially abundant between PWH with and without airflow limitation, analyzed using the DESeq2 package, adjusted for smoking status.


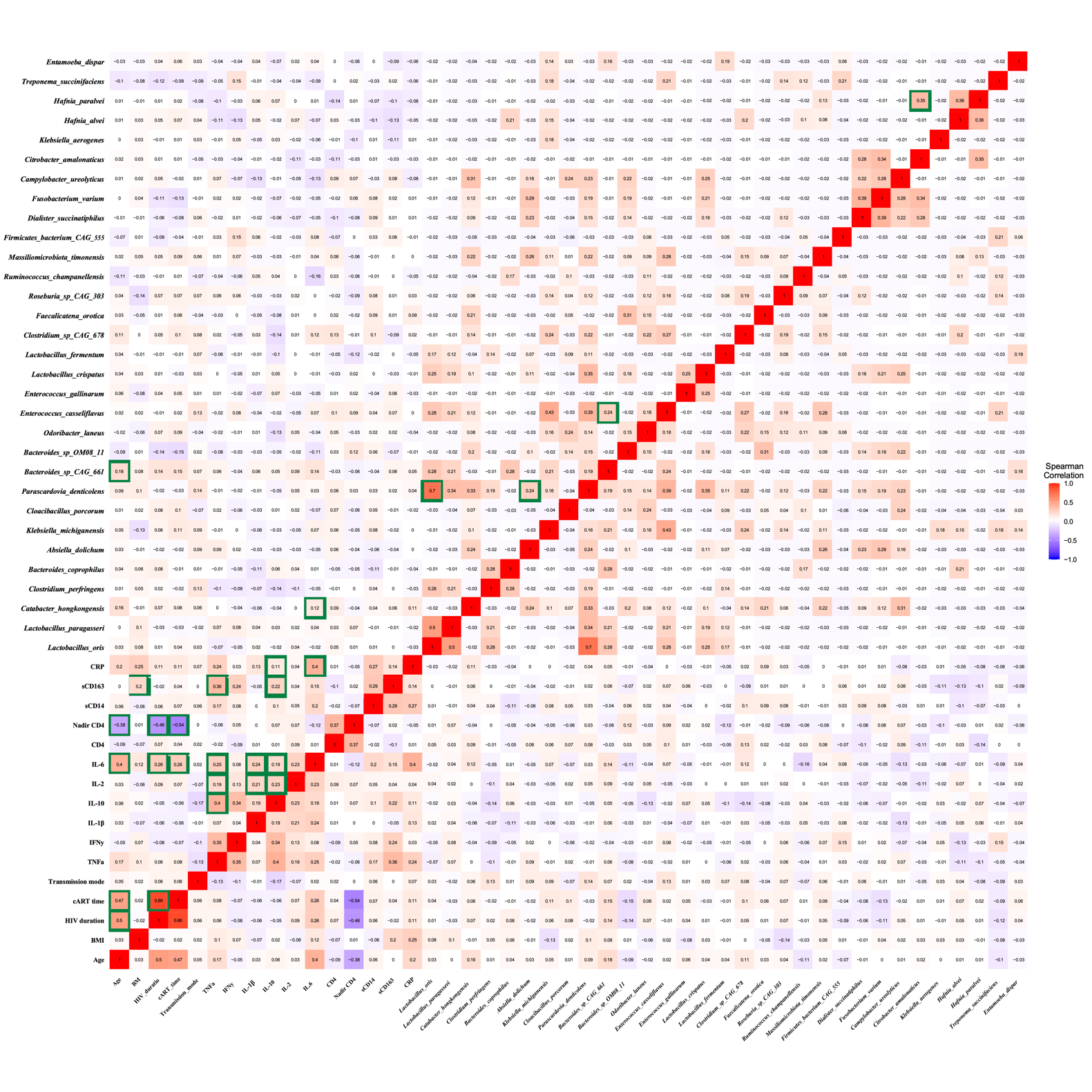
Supplementary **Figure S5**. Correlations of differentially abundant taxa between rapid and normal decliners from DESeq2 analysis, with clinical parameters. Spearman’s correlation values are represented by color gradient as indicated (red is for positive, blue is for negative correlation). Correlations with Benjamini-Hochberg adjusted p<0.05 are highlighted in green squares.


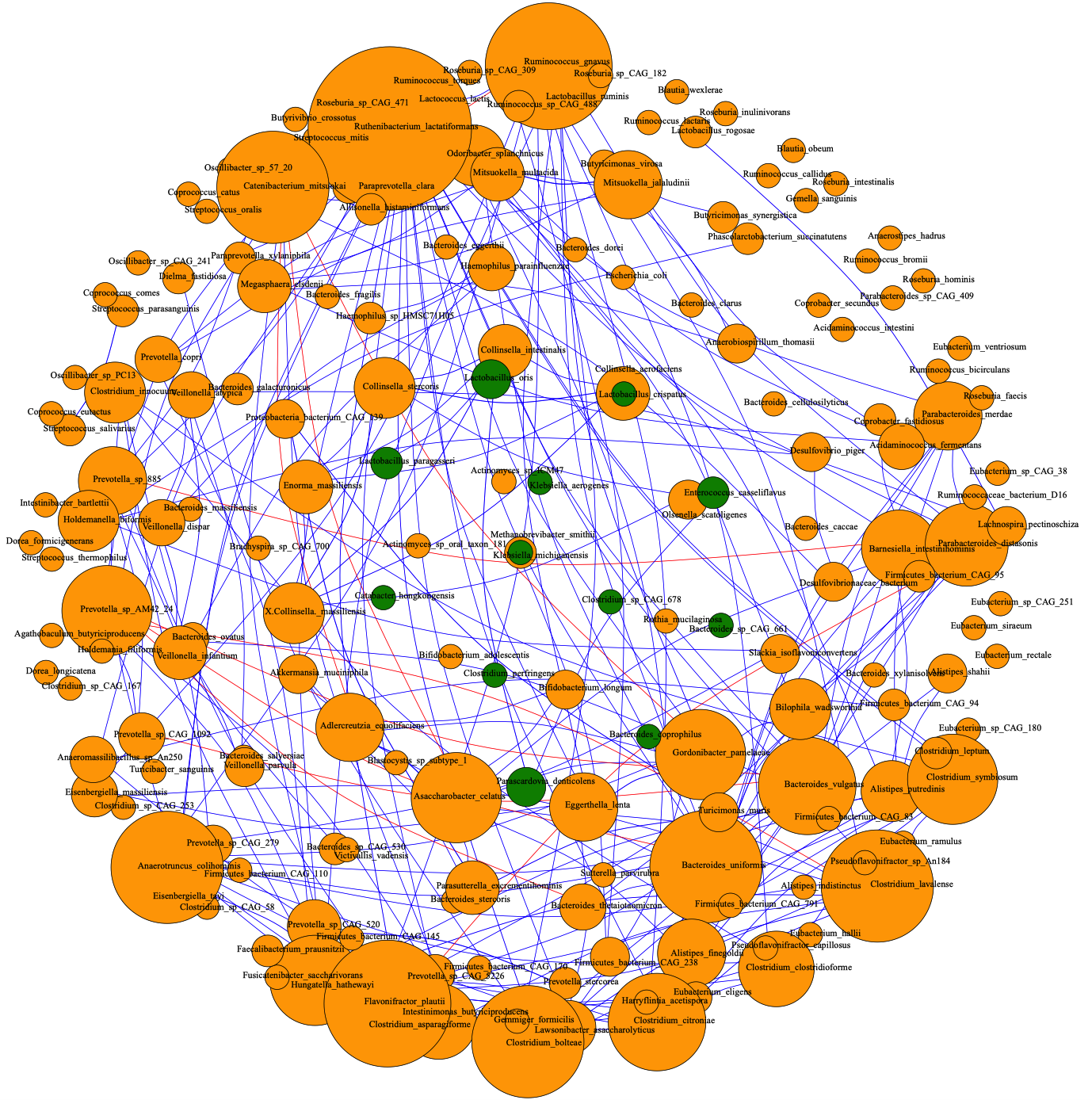
 Figure S6 A

Figure S6 B


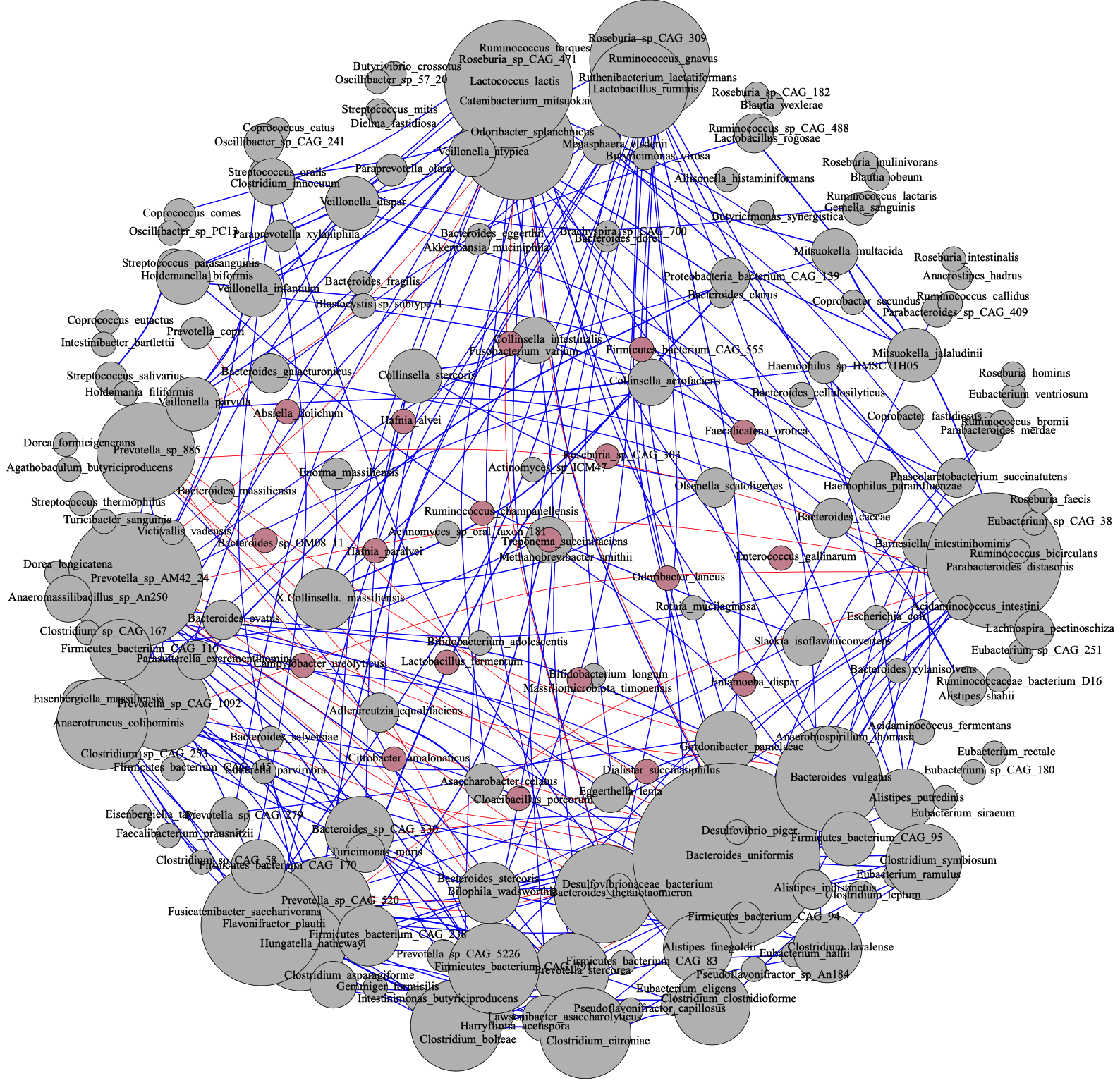


Supplementary **Figure S6**. Co-occurrence network showing the correlations between microbial taxa in rapid decliners (A), and normal decliners (B). Analysis was done on a subset of top abundant microbial taxa, including the differentially abundant taxa between rapid and normal decliners. Edges are shown for strong (Spearman’s correlation coefﬁcient >0.5) and significant (Benjamini-Hochberg adjusted p<0.05) correlations. Blue edges represent positive correlations, while red edges represent negative correlations. Green-colored nodes (A) represent microbial taxa significantly enriched in rapid decliners, and light purple-colored nodes (B) represent microbial taxa enriched in normal decliners.
